# Noninvasive Focused Ultrasound as a Safe Modulator of Calcium-Dependent Neurochemical Signalling in Primary Cortical Cultures

**DOI:** 10.1007/s11064-026-04676-z

**Published:** 2026-01-24

**Authors:** Iqra Bano, Pascal Jorratt, Viera Kútna, Jan Pala, Grygoriy Tsenov

**Affiliations:** 1 Department of Animal Physiology, Faculty of Science, Charles University, Albertov 6, 128 00 Prague, Czech Republic; 2 Department of Experimental Neurobiology, National Institute of Mental Health, Topolová 748, 25067 Klecany, Czech Republic; 3 Department of Physiology and Biochemistry, Faculty of Bioscience, Shaheed Benazir Bhutto University of Veterinary & Animal Sciences, Sakrand, 67210 Pakistan; 4 Third Faculty of Medicine, Charles University, Ruská 87, 100 00 Prague 10, Czech Republic

**Keywords:** Focused ultrasound stimulation, Cortical neurons, Neurochemical signalling, Calcium imaging, Neuromodulation

## Abstract

Focused ultrasound stimulation (FUS) is a promising non-invasive neuromodulation technique that can influence neuronal activity through mechanical stimulation. In this study, primary cortical neurons were isolated from embryonic rat brains and cultured for 14 days in vitro before being divided into Control, FUS 5 V, and FUS 10 V groups. Cells were exposed to low-intensity pulsed FUS (300 kHz, 10 min) using a vertically mounted transducer positioned 5 mm above the culture dish. Post-exposure analyses included cell viability using the MTS assay, total protein quantification by the Bradford method, morphological assessment by Trypan Blue staining, and Fluo-3 AM–based confocal calcium imaging. FUS treatment produced no significant differences in viability or total protein concentration compared with the Control group. Morphological observations confirmed healthy neuronal somata and intact neuritic networks across all groups, with no evidence of cell death or structural damage compared with controls. In contrast, calcium imaging revealed a robust transient elevation in intracellular Ca²⁺ responsiveness when assessed 24 h after FUS exposure, with a significantly higher integrated area under the curve relative to Control. These findings demonstrate that low-intensity FUS safely enhances intracellular calcium signalling while preserving neuronal viability, protein integrity, and morphology, defining a safe acoustic window for non-destructive neuromodulation and providing a framework for mechanistic studies in neurodegenerative disease models.

**Supplementary Information:**

The online version contains supplementary material available at 10.1007/s11064-026-04676-z.

## Introduction

Focused ultrasound (FUS) is an innovative, non-invasive technology that has gained attention for its ability to modulate neuronal activity and enhance therapeutic delivery in various neurological disorders [1, 2]. FUS can locally alter neuronal excitability without requiring surgery by producing low-intensity mechanical pressure waves at frequencies usually higher than 200 kHz that can pass through undamaged tissue [3]. At low intensity, it primarily affects the plasma membrane and mechanosensitive ion channels mechanically, changing membrane permeability and intracellular signalling cascades reversibly [4]. These special qualities have made FUS a viable substitute or addition to well-known brain-stimulation techniques as transcranial magnetic stimulation and deep brain stimulation [5]. In neuronal physiology, calcium (Ca^2+^) signalling plays a key role in controlling gene transcription, cell survival, synaptic transmission, and plasticity. It is now understood that Ca^2+^ homeostasis disturbances are a major early cause of several neurodegenerative illnesses, such as Parkinson’s disease (PD) and Alzheimer’s disease (AD), Amyotrophic lateral sclerosis (ALS), as well as cerebral ischemia (CI) [6]. Therefore, it is of tremendous scientific and clinical interest to develop experimental procedures that can safely and selectively modulate Ca^2+^ flux [7]. At the neurochemical level, intracellular Ca^2+^ fluctuations regulate key signalling molecules such as calmodulin, CaMKII, and CREB, which coordinate neurotransmitter release, synaptic plasticity, and neuronal survival [8]. Mechanosensitive ion channels, including Piezo1, TRPV, and Pannexin-1, have recently been identified as critical transducers of ultrasound-induced mechanical forces that trigger Ca^2+^ entry and activate downstream second-messenger pathways [9]. Understanding these molecular underpinnings is essential for validating FUS as a controlled neuromodulatory tool rather than a nonspecific physical stimulus [1]. According to multiple studies, high-frequency ultrasound can cause brief intracellular Ca^2+^ increases and initiate downstream signalling in a variety of cell types, making FUS a viable choice [10]. However, most published research has been carried out in acute in vivo preparations or immortalized cell lines, where the synaptic architecture and cellular milieu differ from those of mature cortical neurons [11]. This gap highlights the need for biologically relevant models [12]. Primary cortical neurons derived from embryonic rat brain provide an in vitro system that preserves native dendritic morphology, synaptic connectivity, and ion channel composition [13]. To determine if ultrasound can alter neuronal Ca^2+^ signalling without inducing stress reactions or rupturing delicate neuronal networks, it is imperative to investigate the effects of FUS in such cultures [14]. Despite growing interest in ultrasound-based neuromodulation, comprehensive investigations of low-intensity FUS in primary cortical cultures remain limited. To address this gap, the present study examined how low-intensity FUS influences intracellular Ca^2+^ signalling and neuronal stability in primary cortical neurons. By integrating live-cell Ca^2+^ imaging with molecular and morphological analyses in a physiologically relevant in vitro model, we evaluated whether FUS can be applied as a safe and effective neuromodulatory approach. Our findings demonstrate that low-intensity pulsed FUS acts as a precise, non-damaging stimulus capable of modulating Ca^2^-dependent neuronal activity without compromising cell viability or protein integrity. These results fill a critical gap in current FUS research and provide a cellular foundation for future investigations aimed at translating noninvasive neuromodulation strategies toward neuroprotective or disease-modifying applications in vivo.

## Materials and Methods

### Isolation of Primary Cortical Neurons from Rat Embryos

The experimental procedures involving animals were approved by the Ethics Committee of the National Institute of Mental Health. The dissection and isolation of cortical cells from rat embryos (day 18) was performed by following the Jorratt et al. (2022) [15]. Briefly, pregnant Wistar rats (approximately 3–4 months old, 250–300 g body weight) were sacrificed via cervical dislocation, in accordance with established ethical guidelines for animal research. Embryonic brains were carefully extracted using sterilized, autoclaved surgical instruments under aseptic conditions to prevent contamination. The cortical regions were isolated in a 15 ml conical tube and washed thrice with ice-cold Hank’s Balanced Salt Solution (HBSS; Thermo Fisher Scientific, Waltham, MA, USA; Cat. No. 14175095). Then, they were transferred to a 1.5 mL microcentrifuge tube and triturated using a 0.9 mm needle (B. Braun, 4657519) and a 0.45 mm needle (B. Braun, 4657683) three times each, at a pace of one drop per second. The tissue suspension was filtered through a 100-µm pore nylon filter to remove undissociated tissue fragments and debris. The filtered suspension was collected into a seeding medium composed of Dulbecco’s Modified Eagle Medium (DMEM; Biowest, Nuaillé, France; Cat. No. L0104-500), supplemented with 10% fetal bovine serum (FBS; Biowest, Nuaillé, France; Cat. No. S1810) and 1% penicillin-streptomycin (1%; Thermo Fisher Scientific, Waltham, MA, USA; Cat. No. 15070063), to provide essential nutrients and maintain sterility for optimal cell viability and growth. Cells were counted using a hemocytometer, and dilutions were prepared according to the experiment: 125,000 cells/cm² were plated for the MTS assay and around 50,000 cells/cm^2^ for Ca^2+^ imaging in 35 mm petri dishes (Cellvis, Mountain View, CA, USA; Cat. No. D35-14-1.5.5-N) coated with Poly-L-lysine. Cultures were incubated at 37 °C and 5% CO_2_ for 24 h. The next day, the seeding medium was replaced with a growth medium, which contained Neuronal medium with 1% penicillin-streptomycin (1%; Thermo Fisher Scientific, Waltham, MA, USA; Cat. No. 15070063), 2 mM L-Glutamine (Thermo Fisher Scientific, Waltham, MA, USA; Cat. No. 25030149), and 2% B27™ serum-free supplement (Thermo Fisher Scientific, Waltham, MA, USA; Cat. No. A3582801). Every 4–5 days, the growth medium was replaced with a fresh medium.

### Application of FUS

**Fig. 1:**
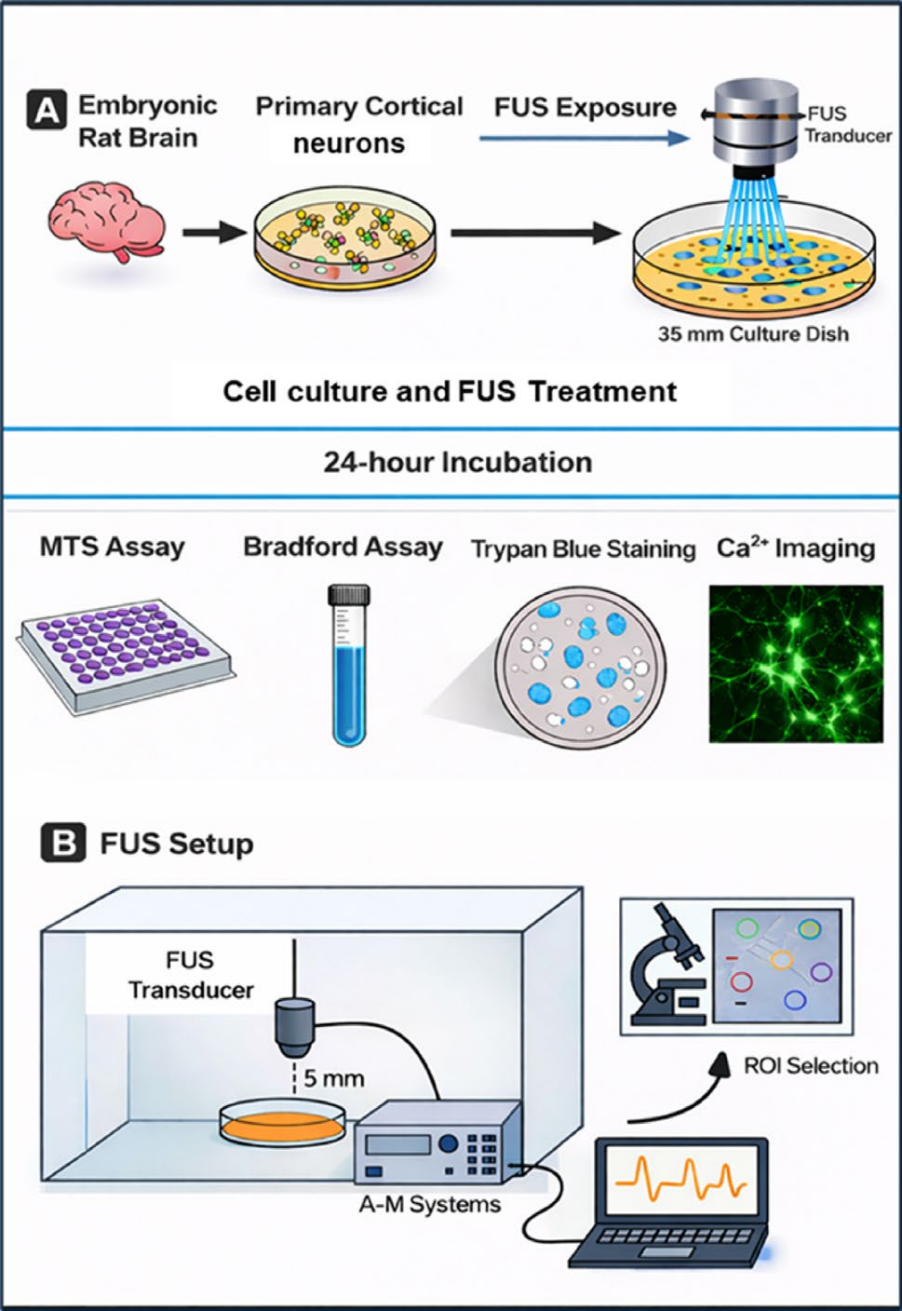
Experimental schematic of FUS in primary cortical neurons. **A** Primary cortical neurons (DIV14) cultured in 35 mm dishes were exposed to low-intensity pulsed FUS (300 kHz, 10 min; 5–10 V drive voltage) using a transducer positioned 5 mm above the culture dish. Cell viability, protein content, morphology, and intracellular Ca^2+^ signaling were assessed 24 h after FUS exposure. **B** FUS stimulation apparatus and Ca^2+^ imaging workflow. A 300 kHz transducer was positioned 5 mm above the culture dish containing primary cortical neurons in pink medium, maintained at 37 °C inside a sterile laminar hood. The transducer was driven by an A-M Systems Model 4100 isolated high-power stimulator connected to a monophasic waveform generator and data-acquisition laptop. The insect illustrates regions of interest (ROIs) selection during confocal Ca^2+^ imaging, where individual somata were analyzed for fluorescence intensity changes following FUS exposure. All ROIs used for functional analysis were selected from areas directly exposed to the ultrasound field

FUS was applied on day 14 in vitro, and the grown cells were divided into two experimental groups named Control and FUS. The Control group did not receive any ultrasound stimulation. In contrast, the FUS group was subjected to FUS at +5 V and +10 V for 10 min using a 300 kHz custom immersion-type transducer compatible with the A-M Systems 4100 High-Power Stimulator. Stimulation strength is reported in terms of applied electrical drive voltage (+5 V and +10 V), which serves as a relative measure specific to this experimental setup, as direct acoustic pressure or intensity calibration of the transducer was not performed. The transducer was sterilized before use through a combination of 70% ethanol treatment and 40 min of UV light exposure. During the experiments, the transducer was positioned vertically (at a 90° orientation) at a fixed distance of 5 mm from the base of the Petri dish. At this distance, the ultrasound field uniformly covered the entire 35-mm culture area; therefore, all imaged cells were located within the insonated region. Hence, under these conditions, the ultrasound beam encompassed the entire culture area, and all subsequent cell viability assays and Ca^2+^ imaging analyses were performed on cells located within the insonated region. During confocal imaging, specific ROIs were manually selected in each FUS-exposed dish to quantify intracellular Ca^2+^ responses. The stimulation parameters included a 300 kHz monophasic pulse with a duration of 0.5 s, a train burst count of 30, and a period of 20 s (Table 1). During stimulation, petri dishes were maintained at 37 °C using a plate heater, and the experimental setup was housed within a laminar flow hood to ensure sterility. After each stimulation session, the transducer was removed, sanitized with 70% ethanol, and reused for subsequent cultures. Following FUS treatment, cells were returned to the incubator for 24 h. In the Control group, cells underwent the same process of transducer submersion and withdrawal without exposure to FUS, ensuring consistency across experimental conditions (Fig. 1).

**Table 1:** FUS experimental protocol and parameter settings programmed on the A-M systems (Sequim, WA, USA) model 4100 isolated High-Power stimulator

FUS device parameters	Details
Train burst quantity	30
Train burst period	20 s
Train burst duration	0.5 s
Pulse frequency	300 kHz
Pulse quantity	150,000
Pulse duration	Half of the event period
Pulse amplitude	+5 V and +10 V
Pulse type	Monophasic
Event period	Defined by a 300 kHz frequency
Event duration	Repeats within each 0.5 s train burst.

### Total Protein Quantification

Following FUS stimulation and incubation for 24 h, cell lysates from each treatment group (Control, FUS 5 V, and FUS 10 V) were collected for total protein quantification. The culture medium was removed, and cells were rinsed twice with cold phosphate-buffered saline (PBS). Neuronal lysates were prepared by adding ice-cold radioimmunoprecipitation assay (RIPA buffer; MilliporeSigma, Burlington, MA, USA; Cat. No. 20–188) and incubating on ice for 20 min with intermittent vortexing. The lysates were centrifuged at 12,000 × g for 10 min at 4 °C, and the supernatants were used for protein measurement. Protein concentration was determined using the Bradford protein assay (Bio-Rad Protein Assay Kit, USA) with bovine serum albumin (BSA; Sigma-Aldrich, St. Louis, MO, USA; Cat. No. A9647) as the standard. A series of BSA standards (0–1.5 mg/mL) was prepared to generate a calibration curve. For each sample, 10 µL of diluted lysate was mixed with 200 µL of Bradford reagent in a 96-well plate, incubated for 5 min at room temperature, and the absorbance was recorded at 595 nm using a microplate reader (Tecan Infinite M200 Pro). All samples were analyzed in triplicate, and protein concentrations were calculated from the BSA standard curve, accounting for the dilution factor, and expressed as µg/mL.

### MTS Cell Viability Assay

Cell viability was evaluated using the 3-(4,5-dimethylthiazol-2-yl)−5-(3-carboxymethoxyphenyl)−2-(4-sulfophenyl)−2 H-tetrazolium (MTS; Thermo Fisher Scientific, Waltham, MA, USA; Cat. No. L11939.03), also known as the MTS colorimetric assay, which is based on the reduction of the MTS tetrazolium compound to generate a formazan product within living cells. Cultures were treated with FUS for 10 min and later incubated for 24 h at 37 °C. After 24 h, 10 µL of MTS solution was added to 100 µL of growth medium and incubated for 2 h at 37 °C with 5% CO_2_. Triton was used as a positive control. After shaking for 20 s, the optical density (OD) was measured at 490 nm using a plate reader (Tecan Infinite M200 Pro). Data was obtained from 5 biological replicas and expressed as a percentage of cell viability using the formula: (OD sample/OD control) × 100. Additionally, trypan blue was used to visualize cell viability in cultures. The growth medium was replaced with 0.02% trypan blue (Gibco, Thermo Fisher Scientific, USA: 15250061) in PBS for 10 min and then washed three times with PBS for 5 min each. Pictures were taken under the inverted phase contrast microscopy.

### Ca^2+^ Imaging and Confocal Microscopy

For Ca^2+^ imaging, primary cortical neurons were divided into Control and FUS-treated groups. Ca^2+^ imaging was performed using the 10 V condition only (selected as the higher stimulation setting); therefore, voltage-dependent differences in Ca^2+^ responses between 5 V and 10 V are not reported. Cells in the FUS-treated group were exposed to low-intensity pulsed ultrasound at 10 V for 10 min, while control cells did not receive FUS but were kept under identical conditions inside the biosafety cabinet for the same duration of time. All Ca^2+^ imaging experiments were performed 24 h after FUS exposure (offline assessment); no real-time Ca^2+^ recordings were acquired during FUS delivery. After FUS treatment, both groups were incubated with the Ca^2+^ -sensitive fluorescent dye Fluo-3 AM (Thermo Fisher Scientific, Waltham, MA, USA; Cat. No. F1241) for 1 h at 37 °C in the dark. Following incubation, cells were washed three times with PBS and allowed to stand for 30 min to enable complete de-esterification of the dye. Fresh media was added, and Ca^2+^ imaging was performed using a Leica TCS SP8X confocal microscope. During live-cell recording, 200 µL of 1 mM KCl was added at the 60-second mark to induce Ca^2+^ influx through voltage-gated Ca^2+^ channels, as a positive control for neuronal responsiveness. Time-lapse recordings were acquired continuously for 8 min to capture dynamic changes in intracellular Ca^2+^ levels, expressed as relative fluorescence intensity (F/F₀).

## Statistical Analysis

All quantitative experiments were performed in triplicate using at least three independent primary neuronal preparations (*n* = 3). Data are presented as mean ± standard error of mean (SEM). Differences in total protein concentration and cell viability among the Control, FUS 5 V, and FUS 10 V groups were analyzed using one-way analysis of variance (ANOVA) followed by Tukey’s post hoc tests. Western blot densitometry values, normalized to total protein, were also compared using one-way ANOVA. For Ca^2+^ imaging analyses, each experimental condition (Control and FUS 10 V) was recorded once per culture dish, and multiple ROIs were analyzed within each field to quantify fluorescence intensity over time. Ca^2+^ responses were expressed as normalized fluorescence ratios (F/F₀), and the integrated area under the curve (AUC) was compared between the Control and FUS-treated groups using an unpaired two-tailed Student’s t-test. In all analyses, a *p* < 0.05 was considered statistically significant.

## Results

### Effects of FUS on Cell Viability and Total Protein Content

**Fig. 2:**
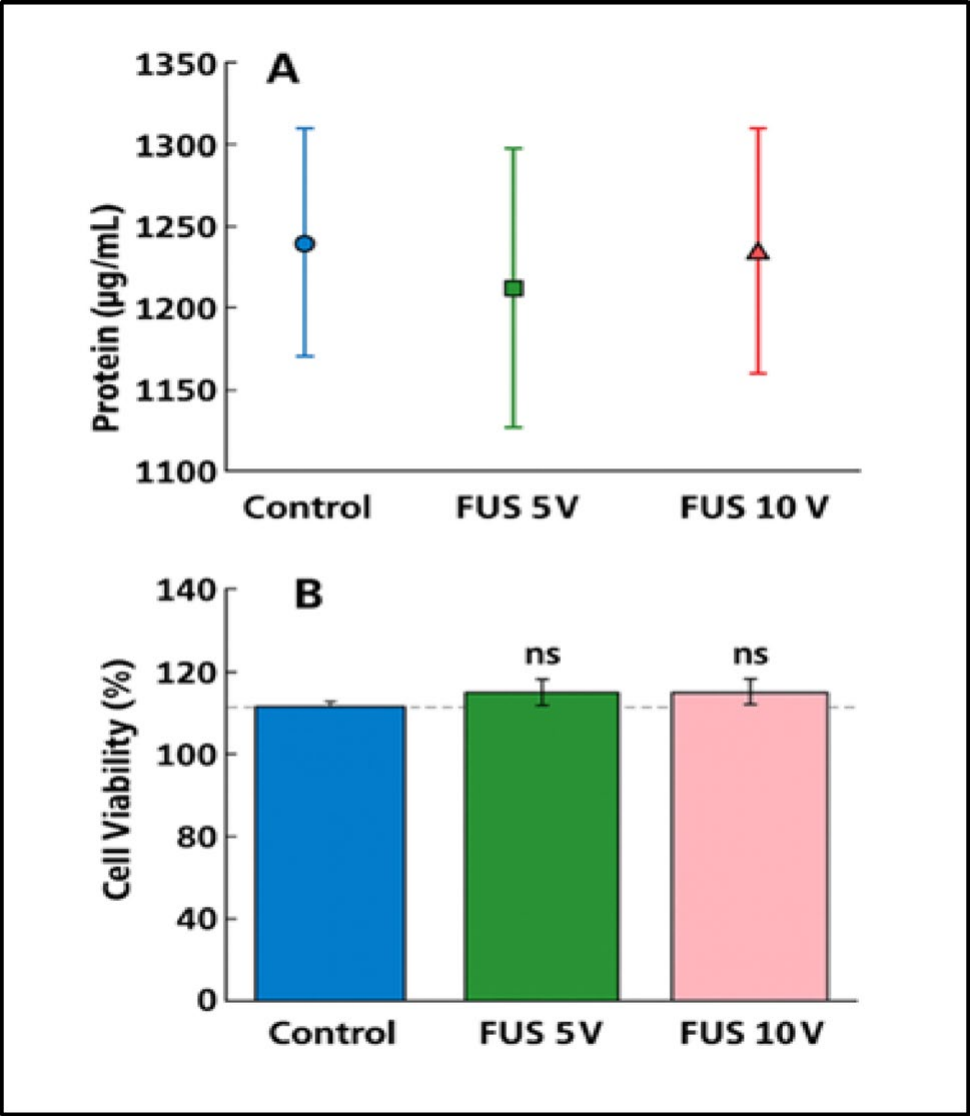
Effects of FUS on total protein content and cell viability in primary cortical neurons. **A** Total protein concentration (µg/mL) measured in Control, FUS 5 V, and FUS 10 V groups. Data are presented as mean ± S.E.M. (*n* = 3). No significant differences were detected among groups (one-way ANOVA, F (2,12) = 0.52, *p* = 0.61). **B** Cell viability (%) assessed by the MTS assay 24 h after FUS exposure. Viability values are expressed relative to the untreated Control (dashed line = 100%). Blue, green, and pink bars represent Control, FUS 5 V, and FUS 10 V groups, respectively. One-way ANOVA indicated an overall group effect (F (2,129) = 4.48, *p* = 0.013); however, post hoc analysis using Tukey’s test revealed no significant pairwise differences between groups (ns)

Exposure of Primary cortical neurons to low-intensity pulsed FUS at drive voltages of 5 and 10 V for 10 min did not induce detectable cytotoxic effects. The total protein concentration (Fig. 2A) did not differ significantly among the Controls, FUS 5 V, and FUS 10 V groups (one-way ANOVA, F (2,12) = 0.52, *P* = 0.61), indicating that FUS exposure did not alter overall protein content or compromise cellular metabolic integrity. Similarly, the cell viability assessed by MTS assay 24 h after FUS exposure remained comparable to untreated controls across all groups (Fig. 2B). Although one-way ANOVA revealed an overall group effect (F (2,129) = 4.48, *p* = 0.013), post hoc analysis using Tukey’s test showed non-significant differences between Control, FUS 5 V, and FUS 10 V conditions. Together, all these results demonstrate that the FUS parameters used in this study do not adversely affect neuronal viability or protein content, supporting the biological safety of selected stimulation conditions for subsequent functional analysis.

### Morphological Assessment of Neuronal Integrity Following FUS Stimulation

**Fig. 3:**
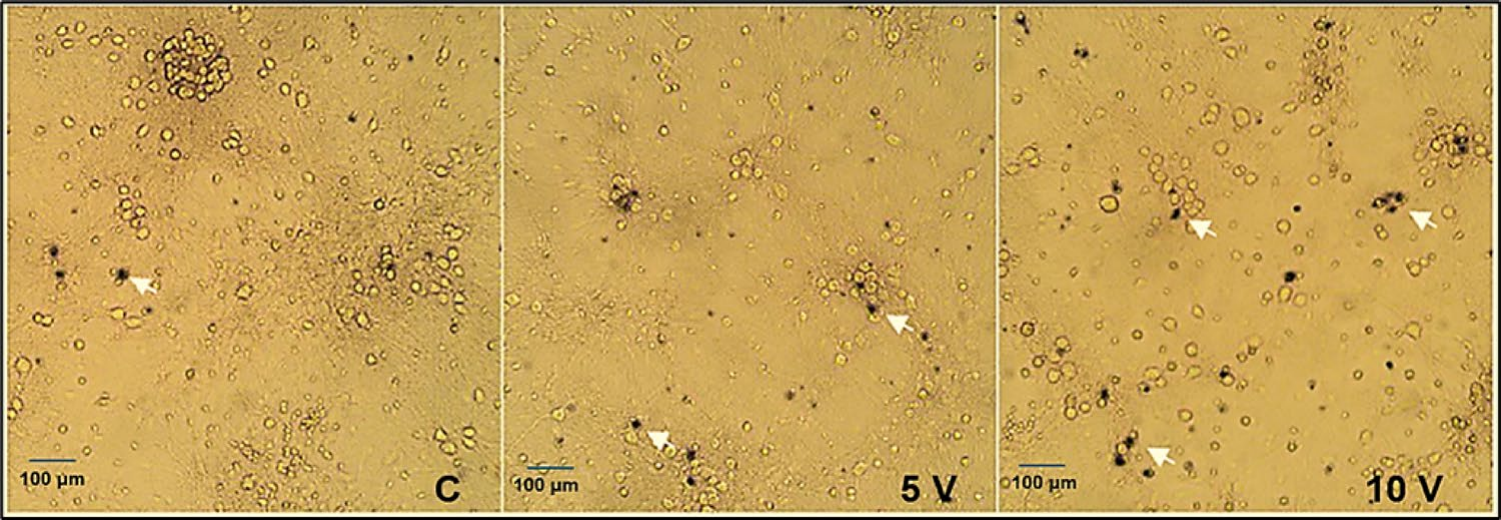
Morphological assessment of primary cortical neurons after FUS stimulation using Trypan Blue staining. Representative bright-field micrographs of primary cortical neurons stained with Trypan Blue to evaluate cell viability and morphology 24 h after treatment. Panels show untreated control cells, cells exposed to 5 V FUS, and cells exposed to 10 V FUS. White arrows indicate viable neuronal cell bodies and intact neuritic processes. Cells in all groups displayed preserved morphology with no signs of shrinkage, membrane blebbing, or neurite fragmentation, indicating that low-intensity pulsed FUS did not cause overt structural damage or a pronounced increase in non-viable cells compared with control cultures. Scale bar = 100 μm

Bright-field micrographs of Trypan Blue-stained neurons (Fig. 3) revealed preserved cell morphology across all experimental conditions. Control, FUS 5 V, and FUS 10 V cultures displayed healthy neuronal somata with clearly defined processes and intact neuritic networks. No signs of cell shrinkage, membrane blebbing, or neurite fragmentation were detected. Although Trypan Blue-positive (non-viable) cells were observed in all experimental groups, no marked increase in non-viable cells was evident in FUS-treated cultures compared with controls. These observations are consistent with quantitative viability data, indicating that low-intensity FUS does not result in a detectable increase in morphological damage or plasma-membrane compromise relative to control cultures.

### Baseline Ca^2+^ Dynamics Under Control Conditions

**Fig. 4:**
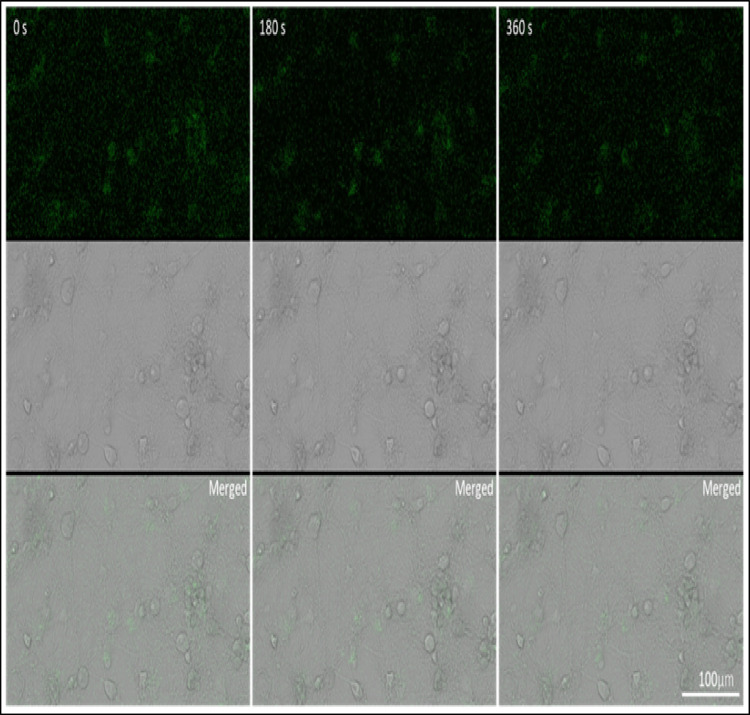
Ca^2+^ imaging of primary cortical neurons under control conditions. The appearance of primary cortical neurons following incubation with the Ca^2+^ indicator Fluo-3 AM and during baseline confocal imaging. Representative time-lapse frames show fluorescence at 0 s, 180 s, and 360 s (top row), corresponding bright-field images (middle row), and merged overlays (bottom row). The cells maintained stable morphology and baseline fluorescence intensity throughout the recording, demonstrating normal physiological conditions after dye loading and before FUS. Scale bar = 100 μm

To establish baseline Ca^2+^ activity, primary cortical neurons were loaded with the Ca^2+^ -sensitive dye Fluo-3 AM and imaged under control conditions without FUS exposure (Fig. 4). Time-lapse recordings acquired over 360 s showed stable intracellular fluorescence with no spontaneous Ca^2+^ transients or oscillations, indicating physiological stability and absence of mechanical or chemical activation. The cells maintained intact morphology and consistent fluorescence distribution during imaging, confirming that the dye loading and imaging procedures themselves did not perturb neuronal activity.

### FUS-Induced Intracellular Ca^2+^ Signalling

**Fig. 5:**
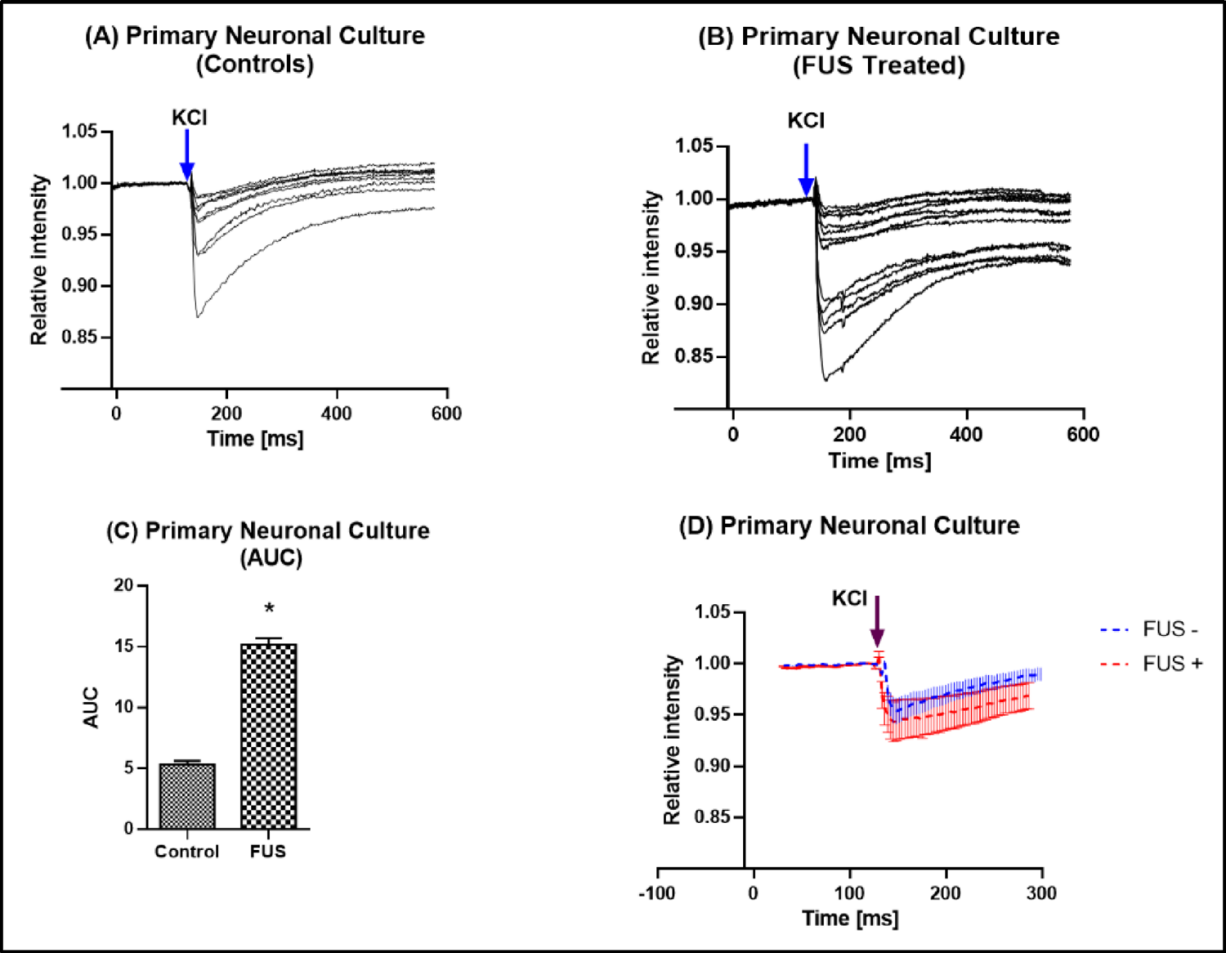
Representative Ca^2+^ fluorescence traces (F/F₀) from Fluo-3 AM–loaded primary cortical neurons recorded under control and FUS conditions. Blue and red traces represent control and FUS-treated cells, respectively. Neuronal depolarization was induced by KCl application (arrow), evoking a transient increase in intracellular Ca^2+^ levels. Neurons previously exposed to FUS exhibited enhanced Ca^2+^ responsiveness to KCl stimulation, followed by a gradual recovery toward baseline. Traces shown are representative examples obtained from individual neuronal somata and illustrate typical Ca^2+^ responses following FUS exposure

Following FUS stimulation at 10 V, a marked increase in intracellular Ca^2+^ fluorescence was observed in Fluo-3 AM-loaded neurons when assessed 24 h after exposure (Fig. 5). The normalized fluorescence ratio (F/F₀) exhibited a sharp transient elevation in intracellular Ca^2+^ responsiveness when assessed 24 h after FUS exposure, followed by gradual recovery toward baseline. Quantitative analysis based on multiple ROIs drawn over individual neuronal somata revealed a significant increase in the integrated AUC compared with control recordings (*p* < 0.001, unpaired two-tailed t-test). This transient but reproducible Ca^2+^ elevation demonstrates that low-intensity pulsed FUS effectively activates neuronal Ca^2+^ signalling without causing structural or metabolic stress.

### ROI-Based Ca^2+^ Response Analysis

**Fig. 6:**
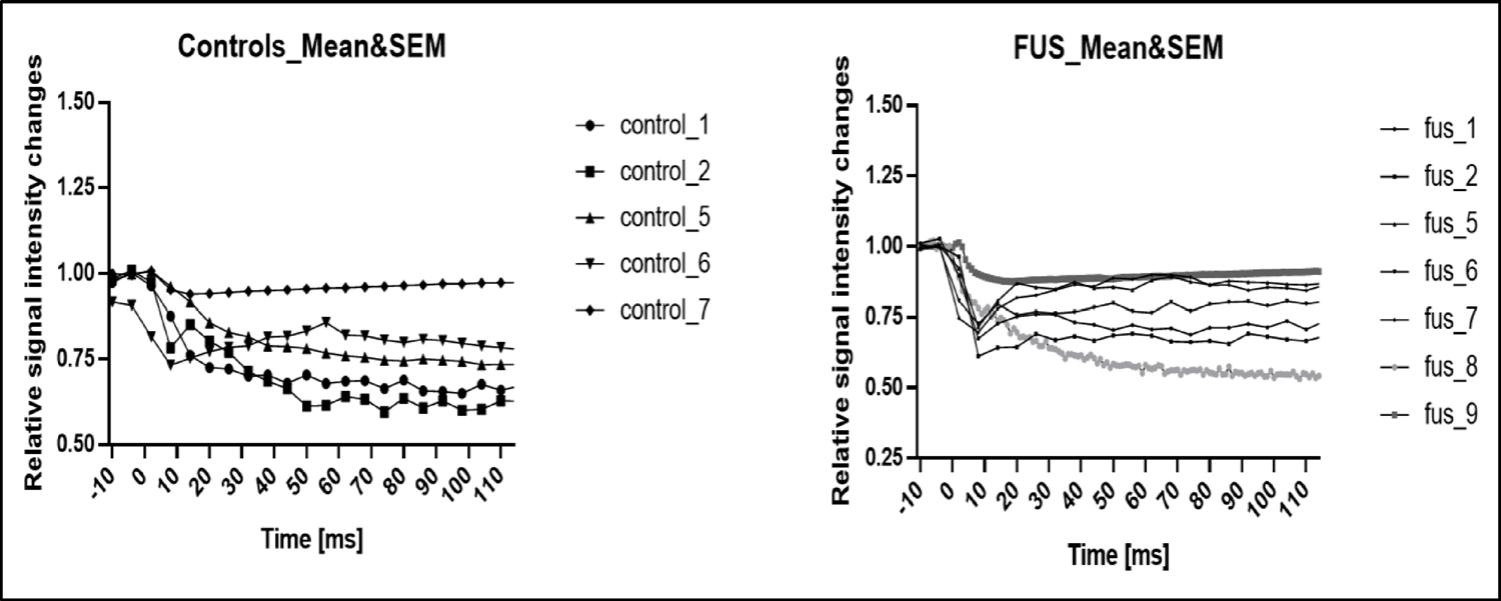
ROI-based Ca^2+^ fluorescence analysis of primary cortical neurons under control and FUS stimulation. Representative ROI-based fluorescence traces (F/F₀) showing intracellular Ca^2+^ dynamics from individual neuronal ROIs under control (gray) and FUS (black) conditions. Each trace represents the mean fluorescence signal from a single ROI selected over neuronal somata. When assessed 24 h after FUS exposure, FUS-treated neurons exhibited a rapid and pronounced increase in intracellular Ca^2+^ fluorescence intensity, followed by a gradual decay toward baseline levels, whereas control neurons showed stable fluorescence throughout the recording period. Quantitative analysis of ROI-based Ca^2+^ responses was performed by calculating the integrated AUC and is presented as mean ± S.E.M. FUS stimulation resulted in a significantly higher AUC compared with Control, indicating enhanced Ca^2+^ responsiveness in FUS-treated neurons

Detailed ROI-based analysis confirmed that FUS stimulation elicited robust and localized Ca^2+^ transients within individual neuronal regions (Fig. 6). Each ROI trace represented Ca^2+^ dynamics from a single neuronal soma or dendritic compartment. While control neurons maintained stable baseline fluorescence throughout the recording, FUS-treated cells exhibited rapid, ROI-specific increases in Ca^2+^ intensity immediately after stimulation onset, followed by a gradual decay phase. The consistent pattern across multiple ROIs within the same culture validates the reproducibility of the response and indicates that the observed Ca^2+^ influx originates from FUS-driven activation of mechanosensitive ion channels rather than random fluctuations. Collectively, these findings establish low-intensity FUS as a reliable and non-invasive modulator of neuronal Ca^2+^ signalling in vitro.

## Discussion

The present study provides direct neurochemical evidence that FUS can modulate intracellular Ca^2+^ signalling in primary cortical neurons without inducing cytotoxic or structural damage. Unlike previous work limited to immortalized cell lines or acute brain slices, our data reveal that mechanical stimulation through FUS can trigger controlled, transient Ca^2+^ elevations in mature cortical cultures that preserve neuronal viability, protein integrity, and morphology. These findings extend the concept of ultrasound neuromodulation beyond physical excitation to the level of intracellular biochemical signalling, suggesting that FUS can serve as a safe and tunable stimulus for regulating Ca^2^-dependent neuronal pathways involved in neurotransmission, metabolism, and plasticity. Our data demonstrate that low-intensity FUS safely modulates neuronal activity by enhancing intracellular Ca^2+^ signalling without affecting cell viability, total protein content, or morphology in primary cortical neurons. The applied acoustic parameters (300 kHz, 5–10 V, 10 min) were well tolerated, as shown by the absence of cytotoxicity in MTS and Trypan Blue assays and unchanged total protein levels measured by the Bradford method (Fig. 2). These results collectively confirm that FUS exposure under our experimental conditions did not induce metabolic stress or membrane damage, highlighting the biological safety of this protocol. Similar findings have been reported by Blackmore et al. (2019) and Kim et al. (2021), who observed no alterations in neuronal viability or protein synthesis following low-intensity ultrasound stimulation in vitro and in vivo [16, 17]. The stable protein levels detected across the Control, FUS 5 V, and FUS 10 V groups further indicate that FUS did not alter global protein turnover or degradation. This observation aligns with previous work showing that mechanical ultrasound energy, when applied below the cavitation threshold, does not disrupt cytoskeletal or synaptic protein expression [18, 19]. The preserved morphology observed in Trypan Blue-stained neurons supports this interpretation, as all groups maintained healthy somata, intact neuritic networks, and the absence of membrane blebbing or cell shrinkage (Fig. 3). These morphological results strengthen the conclusion that low-intensity FUS exerts mechanical, non-destructive effects on primary neurons. The notion that pulsed, low-intensity FUS has mechanical but non-destructive effects is also supported by earlier research that used similar or slightly higher acoustic intensities and found no disruption of membrane integrity or protein homeostasis in cultured neurons or brain slices [16]. The absence of spontaneous Ca^2+^ activity under baseline conditions in our data indicates that the neuronal cultures were physiologically stable before stimulation, supporting the interpretation that the Ca^2+^ changes observed after FUS exposure are stimulus-dependent rather than arising from inherent cellular activity or imaging-related effects (Fig. 4). As discussed previously, that in neurons and other mechanosensitive cells, piezo1 in particular has been identified as a crucial mechanotransducer [20]. Piezo1 suppression, either genetically or pharmacologically, dramatically reduced ultrasound-induced Ca^2+^ influx and subsequent neuronal activation, according to recent in vivo studies [21, 22]. Similarly, TRP (transient receptor potential) family members, including TRPV and TRPC channels, have been connected to ultrasonic responses in cortical neurons [18] and are well known to mediate mechanically driven Ca^2+^ entry [23]. In this regard, the notable rise in relative fluorescence and AUC seen in our FUS-treated neurons is in line with the quick, mechanically gated opening of TRP and Piezo1 channels, which permits Ca^2+^ influx to be sustained. Intracellular reserves probably add to the sustained Ca^2+^ increase that causes the higher AUC in addition to plasma membrane influx (Fig. 5). Previously, FUS has been shown by Lee and colleagues (2020) to directly trigger Pannexin-1 (PANX1) channels located in the endoplasmic reticulum, which causes the release of Ca^2+^ from internal reserves [24]. Such ER-derived release would enhance the integrated Ca^2+^ signal and prolong the transient’s decay phase, which is remarkably like the prolonged AUC we saw.

Our data of functional Ca^2+^ imaging revealed transient yet reproducible elevations in intracellular Ca^2+^ immediately after FUS application, confirming that the same acoustic parameters that maintained structural integrity were sufficient to trigger neuronal activation. The ROI-based fluorescence analysis revealed a robust increase in Ca^2+^ signal amplitude and AUC compared to non-stimulated controls, demonstrating localized and controlled Ca^2+^ entry (Fig. 6). Mechanistically, these responses are consistent with the activation of mechanosensitive ion channels, including Piezo1, TRPV, and Pannexin-1 (PANX1), which have been shown to mediate ultrasound-induced Ca^2+^ influx in neuronal and non-neuronal cells [24–26]. Moreover, the temporal characteristics of the Ca^2+^ response in our data show a rapid onset followed by a gradual recovery, mirroring previously described FUS-evoked Ca^2+^ transients in cortical neurons [27]. The combination of unaltered protein content preserved cell viability, and strong yet non-lethal Ca^2+^ signalling supports the concept that low-intensity FUS can safely enhance neuronal excitability without inducing damage or altering homeostatic processes. Overall, our findings agree with previous studies demonstrating that controlled FUS exposure can modulate neuronal function safely [16, 17, 21]. Importantly, this study extends those observations to primary cortical neurons, a physiologically relevant model preserving native dendritic and synaptic organization. The concurrent evaluation of metabolic (MTS), structural (Trypan Blue, protein), and functional (Ca^2+^ imaging) endpoints provides comprehensive evidence that FUS is both safe and effective in vitro. In summary, low-intensity pulsed FUS at 300 kHz induces transient, ROI-specific increases in intracellular Ca^2+^ while preserving neuronal viability, protein stability, and morphology. These findings establish a safe acoustic window for future mechanistic studies and support the use of FUS as a noninvasive neuromodulation strategy in neuronal culture systems and disease models. A limitation of the present study is the absence of direct acoustic pressure field calibration; nevertheless, future work should explore the long-term effects of repeated FUS exposure, integrate electrophysiological and high-resolution Ca^2+^ imaging approaches to elucidate underlying mechanosensitive mechanisms, and extend this methodology to disease-relevant and in vivo models to facilitate the translational development of FUS-based neuromodulation.

## Supplementary Information

Below is the link to the electronic supplementary material.

Supplementary Material 1

## Data Availability

Additional resources shall be provided upon inquiry to the corresponding author.
