# Supplementary figures and images for: Noninvasive Focused Ultrasound as a Safe Modulator of Calcium-Dependent Neurochemical Signalling in Primary Cortical Cultures
Source: Neurochem Res. 2026 Jan 24;51(1):61. doi: 10.1007/s11064-026-04676-z (PMC12831675; doi:10.1007/s11064-026-04676-z)

Graphical abstract


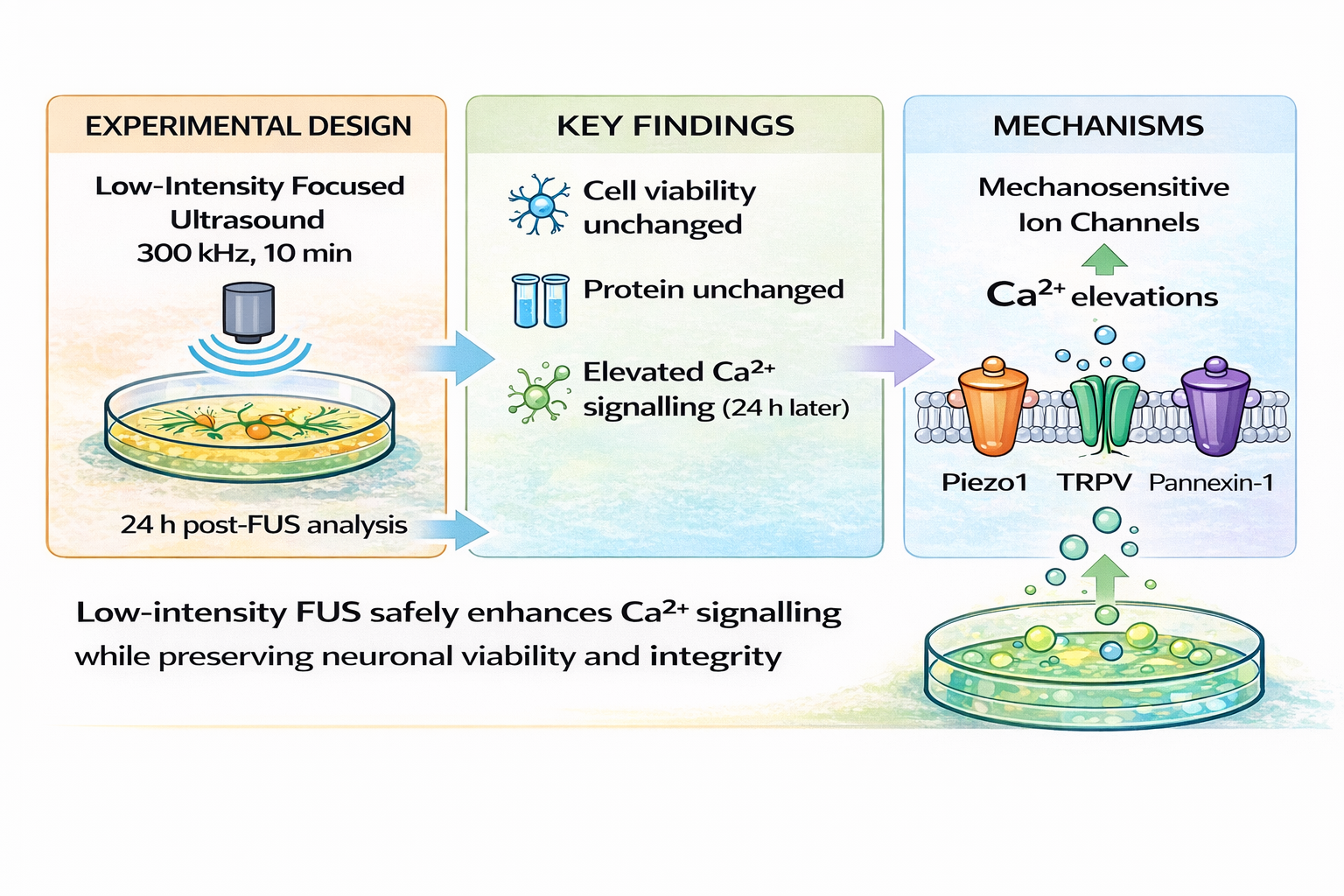

Supplement: Supplementary file 1 — Supplementary Material 1 [file 11064_2026_4676_MOESM1_ESM.docx]
